# Characterization of the Bioactivity and Mechanism of Bactenecin Derivatives Against Food-Pathogens

**DOI:** 10.3389/fmicb.2019.02593

**Published:** 2019-11-05

**Authors:** Changbao Sun, Liya Gu, Muhammad Altaf Hussain, Lijun Chen, Li Lin, Haimei Wang, Shiyue Pang, Chenggang Jiang, Zhanmei Jiang, Juncai Hou

**Affiliations:** ^1^Key Laboratory of Dairy Science, Ministry of Education, College of Food Science, Northeast Agricultural University, Harbin, China; ^2^National Engineering Research Center of Dairy for Maternal and Child Health, Beijing Sanyuan Foods Co., Ltd., Beijing, China; ^3^Harbin Veterinary Research Institute, Chinese Academy of Agricultural Sciences, Harbin, China

**Keywords:** antibacterial peptides, bactenecin derivatives, food-pathogens, bioactivity, mechanism

## Abstract

With the emergence of multidrug-resistant bacteria, antimicrobial peptides (AMPs) are regarded as potential alternatives to traditional antibiotics or chemicals. We designed and synthesized six derivatives of bactenecin (L_2_C_3_V_10_C_11_, RLCRIVVIRVCR), including R_2_F_3_W_10_L_11_ (RRFRIVVIRWLR), R_2_W_3_W_10_R_11_ (RRWRIVVIRWRR), K_2_W_3_V_10_R_11_ (RKWRIVVIRVRR), W_2_R_3_V_10_R_11_ (RWRRIVVIRVRR), W_2_K_3_K_10_R_11_ (RWKRIVVIRKRR), and K_2_R_3_R_10_K_11_ (RKRRIVVIRRKR), by amino acid substitution to increase the net charge and reduce hydrophobicity gradually. The bioactivity and mechanisms of action of the designed peptides were investigated. The results indicated that the antimicrobial activity of the designed peptides was higher than that of bactenecin. The hemolytic activity and cytotoxicity of the designed peptides were significantly lower than those of bactenecin. The designed peptides exhibited a wide range of antimicrobial activity against food-pathogens, particularly peptides K_2_W_3_V_10_R_11_ and W_2_R_3_V_10_R_11_; in addition, the activity was maintained under physiological salt and heat conditions. Mechanism studies indicated that AMPs interacted with negatively charged bacterial cell membranes, resulting in the destruction of cell membrane integrity by increasing membrane permeability and changing transmembrane potential, leading to cell death. The present study suggested that peptides K_2_W_3_V_10_R_11_ and W_2_R_3_V_10_R_11_ exhibited potential as alternatives to traditional antibiotics or chemicals for the treatment of food-pathogens. These findings lead to the development of a potential method for the design of novel AMPs.

## Introduction

The spread of multidrug-resistant microorganisms (superbugs) has drawn increasing interest toward antimicrobial peptides (AMPs), which play important roles in the innate immune systems of many organisms, including vertebrates, invertebrates, plants, and microbes (Tao et al., 2017). AMPs act as the first line of innate defense showing a broad range of antimicrobial activity against food-pathogens (Steckbeck et al., 2014; De et al., 2018). Conventional chemicals and antibiotics mainly inhibit the biosynthesis of certain substances (such as cell wall, proteins, DNA, or RNA) in cells, leading to cell death. Owing to cationic and amphiphilic properties, most AMPs can interact with the negatively charged components such as the phosphate group in lipopolysaccharides (LPS) or teichoic acid on the outer membrane of Gram-negative and Gram-positive bacteria, respectively, disrupting cell membrane integrity, increasing cell membrane permeability, and changing transmembrane potential, leading to cell death (Shai, 2002; Hancock and Sahl, 2006; Tajbakhsh et al., 2018). Thus, this mechanism of action of antimicrobial peptides reduces the probability of bacterial resistance. AMPs are strong candidates to complement or substitute current antimicrobial agents.

Bactenecin, a 12-amino acid AMP found in bovine neutrophils (Romeo et al., 1988), is the smallest known cationic AMP with the sequence RLCRIVVIRVCR-NH_2_, which consist of 4 arginine residues, 2 cysteine residues, and 6 hydrophobic residues. The 2 cysteine residues form a disulfide bond that enabled the antimicrobial peptide molecules to form a folded structure (Wu and Hancock, 1999b). Evidence has shown that bactenecin exhibits extensive antimicrobial activity against bacteria, particularly Gram-negative bacteria (Hilpert et al., 2006), and strong cytotoxicity (Radermacher et al., 1993). However, compared with traditional antibiotics, many natural antimicrobial peptides are not suitable for industrial production because of stability, high extraction costs and biodisponibility problem. Thus, studies are being conducted on the design and synthesis of novel AMPs by using natural antimicrobial peptides as templates. Ding (2013) synthesized bactenecin linear derivatives (Bac8c), which are expected to enhance antibacterial activity and decrease cytotoxicity. The biological activity of antimicrobial peptides is linked to their physical and chemical properties, such as amphipathy, net charge, hydrophobicity, and structural characteristics (Hancock et al., 1995). Recent research has shown that a disulfide bond in bactenecin does not contribute significantly to its antimicrobial activity (Wu and Hancock, 1999a, b). Thus, by amino acid substitution, we designed six linear derivatives of bactenecin acting as templates. The net charge of the designed peptides gradually increased with a decrease in hydrophobicity. The sequence of the derivatives (R_2_F_3_W_10_L_11_, W_2_R_3_V_10_R_11_, K_2_W_3_V_10_R_11_, R_2_W_3_W_10_R_11_, W_2_K_3_K_10_R_11_, and K_2_R_3_R_10_K_11_) was RRFRIVVIRWLR-NH_2_, RWRRIVVIRVRR-NH_2_, RKWRIVVIRVRR-NH_2_, RRWRIVVIRWRR-NH_2_, RWKRIVVIRKRR-NH_2_, and RKRRIVVIRRKR-NH_2_, respectively. We compared the bioactivity of the seven synthesized peptides against food-pathogens and determined the antimicrobial mechanism of AMPs to develop bactenecin derivatives with higher antimicrobial activity, broader antibacterial spectrum, and lower cytotoxicity. This study aims to propose ideas and methods for the application of synthetic AMPs.

## Materials and Methods

### Materials

All peptides were synthesized and purified by GL Biochem Co., Ltd., (Shanghai, China) by chemical solid-phase synthesis. The peptides were purified by reverse-phase high-performance liquid chromatography (RP-HPLC, LC3000, Beijing, China) with a purity of more than 95%. The molecular weight of the peptides was measured by electrospray mass spectroscopy (Model Autoflex, Bruker Daltonics Inc., United States). The peptides were amidated at the C-terminus, dissolved in deionized water to 2.56 mM, and stored at −20°C.

The common food-pathogens (*Salmonella typhimurium* C79-13, *Escherichia coli* ATCC25922, *Salmonella Typhimurium* C77-31, *Salmonella enteric-subspenterica* CMCC47020, *Salmonella enteric- subspenterica* CMCC50071, *Cronobacter sakazakii* ATCC29544, *Salmonella Typhimurium* ATCC14028, *Listeria monocytogenes* CMCC54004, *Bacillus cereus* CMCC6303, *Staphylococcus aureus* CMCC26074, *Escherichia coli* UB1005, *Staphylococcus aureus* ATCC25923) and human red blood cells (hRBCs) were purchased from the Harbin Veterinary Research Institute, Chinese Academy of Agricultural Sciences (Harbin, China). Human umbilical vein endothelial cells EA. hy 926 were purchased from Harbin Medical University. Mueller-Hinton broth (MHB) and Mueller- Hinton agar (MHA) were purchased from AOBOX Biotechnology Co., Ltd. (Beijing, China). Dulbecco’ s modified Eagle’s medium (DMEM) and fetal bovine serum (FBS) were supplied by Gibco Life Technologies Co., Ltd. (New York, United States). All reagents (except for those specially noted) were of analytical grade and purchased from Kermel Chemical Reagent Co., Ltd. (Tianjin, China) and Sigma-Aldrich Co., Ltd. (Shanghai, China).

### Sequence Analysis of Peptides

The primary structure of peptides was analyzed via the website http://www.expasy.org/tools/protparam.html. The hydrophobicity and hydrophobic moment of the peptides were analyzed via the website http://heliquest.ipmc.cnrs.fr/. Helical wheel projections were analyzed via the website http://lbqp.unb.br/NetWheels/.

### Circular Dichroism Spectra

Conformational changes in peptides in different environments were characterized at 25°C with a J-810 spectropolarimeter (Jasco, Tokyo, Japan) using a quartz cell with 1.0 mm path length as described in a previous study (Zhu et al., 2015). The peptide solutions (final concentration of 150 μM) were dissolved in 10 mM phosphate buffered saline (PBS, pH 7.4), 50% trifluoroethanol (TFE), and 30 mM sodium dodecyl sulfate (SDS), respectively. Spectra ranging from 190 nm to 250 nm were recorded at a scanning speed of 10 nm/min and an average of 3 scans was collected for each peptide. The acquired CD spectra were then converted to the mean residue ellipticity by using the following equation: θ_M_ = (θ_obs_ × 1000)/(c × l × n), where θ_M_ is the mean residue ellipticity (deg × cm^2^ × dmol^–1^), θ_obs_ is the observed ellipticity corrected for the buffer at a given wavelength (mdeg), c is the peptide concentration (mM), l is the path length (mm), and n is the number of amino acids.

### Antimicrobial Activity

The antimicrobial activity of each peptide against food-pathogens was characterized by the minimum inhibitory concentration (MIC) (Ma et al., 2013). A logarithmic growth-phase culture of bacteria (1 × 10^6^ CFU/mL) was mixed with peptide solutions (final concentration ranging from 0 μM to 256 μM) in 96-well plates. After the 96-well plates were incubated for 24 h at 37°C, optical dispersion (OD) was measured using a microplate reader (Bio-Rad, ıHercules, CA, United States) at 630 nm. The MICs were determined as the lowest concentration of peptides at which no microbial growth was observed with the unaided eye visually. The broth with bacteria cells was used as the negative control, and un-inoculated broth was used as a positive control. The tests were performed in triplicate using three replicates for each experiment.

### Hemolytic Activity

The hemolytic activity of peptides was determined by measuring the amount of hemoglobin released by human red blood cells (hRBCs) as described in a previous study (Luna-Ramírez et al., 2014). The hRBCs were washed and resuspended in 10 mM PBS (pH 7.4) to attain a dilution of ∼2% heamatocrit. Then, 50 μL of the hRBCs solution was incubated with 50 μL of a serial dilution of peptides dissolved in PBS for 1 h at 37°C. The mixtures were centrifuged, and the absorbance of the supernatant was measured using a microplate reader (Bio-Rad, ıHercules, CA, United States) at 570 nm. The hRBCs treated with PBS (0% hemolysis, A_0_) and 0.01% Triton X–100 (100% hemolysis, A_t_) acted as negative and positive controls, respectively. Melittin was used as a control peptide. The percentage of hemolysis was calculated using the following equation: Hemolysis = [(A × A_0_)/(A_t_ × A_0_)] × 100%.

### Cytotoxicity

The cytotoxicity of the peptides was tested on the EA. hy 926 cell line by using the Cell Counting Kit-8 (CCK-8, Dojindo, Japan) as described in a previous study (Sánchez-Gómez et al., 2015). The EA. Hy 926 cells cultured in the logarithmic growth -phase was then diluted with PBS to a single cell suspension. A 100 μL cell suspension (2 × 10^5^ cells) was added to 96-well plates and cultured in an incubator with a 5% CO_2_ atmosphere at 37°C. The cell culture medium was removed, and the two-fold serial dilutions of peptides prepared using DMEM with 5% FBS were added to the 96-well plates. After 24 h, 100 μL DMEM with 10% CCK-8 was added to the 96-well plates and then cultured for 4 h. The absorbance was measured at 492 nm by using a microplate reader. DMEM served as a positive control and cells mixed with DMEM without peptides as a negative control. Melittin was employed as a control peptide.

### Stability

To evaluate the effect of different physiological conditions on the stability of peptides, we determined the salt stability, thermal stability, and enzymatic stability as described in a previous study (Dong, 2013; Sun et al., 2018). *Escherichia coli* ATCC25922 grown in the logarithmic phase (1 × 10^6^ CFU/mL) was exposed to each peptide at different salt concentrations (150 mM NaCl, 4.5 mM KCl, 6 μM NH_4_Cl, 1 mM MgCl_2_, 8 μM ZnCl_2_, 2 mM CaCl_2_, and 4 μM FeCl_3_). The peptides were treated for 1 h at 100°C to test for thermal stability. The peptides were treated with trypsin, pepsin, papain and protease k at a concentration of 1 mg/mL for 1 h at 37°C to test for enzyme stability. The control group consisted of untreated peptides. The subsequent assay was consistent with the MIC assay.

### Antibacterial Mechanism Study

#### Outer Membrane Permeability

The effect of peptides on the bacterial outer -membrane permeability was characterized by measuring the uptake of *N*-phenyl-1-naphthylamine (NPN) as described in a previous study (Zhu et al., 2014). In short, *Escherichia coli* UB1005 grown in the logarithmic phase was centrifuged at 2000 *g* and resuspended to an OD_600 nm_ of 0.2 in 5 mM of 4−(2-hydroxyethyl) piperazine-1-ethane sulfonic acid (HEPES, pH 7.2) with 5 mM glucose. Subsequently, 2 mL of bacterial suspension was mixed with NPN (1 mM), and the background fluorescence (F_0_) was recorded (excitation λ = 350 nm, emission λ = 420 nm) using an F-4500 fluorescence spectrophotometer (Hitachi, Tokyo, Japan). The peptides with final concentrations ranging from 1 to 16 μM were then added to the bacterial suspension in a quartz cuvette. The fluorescence (F_0__bs_) was recorded over time until no further increase was observed. Polymyxin B (F_100_) acted as a positive control for the test. The percent uptake of NPN was calculated using the following equation: NPN = (F_0__bs_ − F_0_)/(F_100_ − F_0_) × 100%.

#### Inner Membrane Permeability

The effect of peptides on the bacterial cell inner membrane permeability was characterized by measuring the uptake of *O*-nitrophenyl-β-D–galactopyranoside (ONPG) as described in a previous study (Arias et al., 2014). *Escherichia coli* UB1005 grown in the logarithmic phase was centrifuged at 2000 *g* and resuspended to an OD_600 nm_ of 0.05 in PBS (5 mM, pH 7.4) with 1.5 mM ONPG. The peptides (at 1 × MIC) were then mixed with bacterial suspension. The absorbance was recorded at 420 nm every 2 min for 0 ∼ 40 min.

#### Cytoplasmic Membrane Depolarization

The effect of peptides on the cytoplasmic membrane depolarization of *Escherichia coli* UB1005 was determined using the membrane potential-sensitive fluorescent dye diSC_3_-5 as described in a previous study (Shao et al., 2018). *Escherichia coli* UB1005 grown in the logarithmic phase was centrifuged at 2000 *g* and resuspended to an OD_600 nm_ of 0.05 in 5 mM HEPES (pH 7.2) with 20 mM of glucose and 0.1 M KCl. The bacterial suspensions were then mixed with 0.4 μM diSC_3_-5 and incubated for 1 h at 37°C. Up to 2 mL of bacterial suspension with peptides (at 1 × MIC) was placed in a quartz cuvette. Fluorescence intensity was monitored in the 0 ∼ 300 s range with an excitation wavelength of 622 nm and an emission wavelength of 670 nm.

#### Flow Cytometry

The destruction of the bacterial cell membrane was measured using *Escherichia coli* ATCC25922 by flow cytometry (Mishra et al., 2013). *Escherichia coli* ATCC25922 grown in the logarithmic phase was centrifuged at 2000 *g* and resuspended to an OD_600 nm_ of 0.2 in PBS (pH 7.4). The peptides at 1× MIC were added to the bacterial suspension and incubated in propidium iodide (PI) with a final concentration of 10 mg/mL for 30 min at 4°C. The unbound dye was then washed with PBS. The data were recorded by fluorescence-activated cell sorting (Becton Dickinson, Franklin Lakes, NJ, United States).

#### Electron Microscopic Characterization

Damage to the integrity of bacterial cell membranes caused by peptides was characterized by scanning electron microscopy (SEM, Hitachi, Japan) and transmission electron microscopy (TEM, Hitachi, Tokyo, Japan) as described in a previous study (Tan et al., 2017). *Escherichia coli* ATCC25922 grown in the logarithmic -phase was centrifuged at 2000 *g* and resuspended to an OD_600 nm_ of 0.2 in PBS (pH 7.4). Peptides at 1 × MIC were added to the bacterial suspension and incubated for 2 h at 37°C in a shaker. The cells were washed three times with PBS and added into 1 mL of 3% glutaraldehyde, placed in a 4°C refrigerator for the night. The cells were then dehydrated with different concentrations of ethanol and tertiary butanol. In the preparation of the SEM sample, the cells were freeze-dried and coated with gold/palladium and then observed using the S-4800 SEM instrument. In the preparation of the TEM sample, the cells were incubated overnight in epoxy resin and acetone and then sectioned, stained, and observed using the H-7650 TEM instrument.

### Statistical Analysis

All tests were conducted at least three times. The data were statistically analyzed using SPSS 20.0 via ANOVA and presented as the means ± standard error.

## Results

### Sequence Analysis of Peptides

**FIGURE 1:**
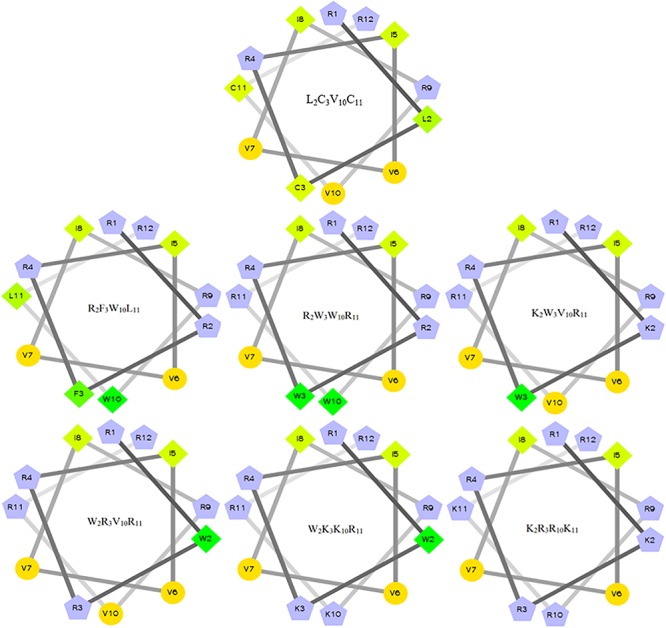
Helical wheel projections of the peptides. By default, the output presents the hydrophilic residues as circles, hydrophobic residues as diamonds, potentially positively charged as pentagons. Hydrophobicity is color-coded as well: the most hydrophobic residue is green, and the amount of green decreases proportionally to the hydrophobicity, with zero hydrophobicity coded as yellow. Hydrophilic residues are coded red with pure red being the most hydrophilic (uncharged) residue, and the amount of red decreasing proportionally to the hydrophilicity. The potentially charged residues are light blue.

The characteristics of the primary structural properties of native and designed peptides are listed in Table 1. A measured molecular weight value of each peptide was in very close agreement with its theoretical value, indicating the successful synthesis of the peptides. Table 1 reveals that all peptides exhibit cationic character, and the net charge is increased gradually in the following order: L_2_C_3_V_10_C_11_ < R_2_F_3_W_10_L_11_ < R_2_W_3_W_10_R_11_ = K_2_W_3_V_10_R_11_ = W_2_R_3_V_10_R_11_ < W_2_K_3_K_10_R_11_ < K_2_R_3_R_10_K_11_. Conversely, the hydrophobicity of the peptides is decreased gradually with the increase in positive charge. The hydrophobicity of the peptides was determined theoretically and experimentally. The mean hydrophobicity (H) was determined as the theoretical hydrophobicity of the peptides and calculated as the total hydrophobicity divided by the number of residues. The experimental hydrophobicity of the peptides was quantified using RP-HPLC retention time (Rt). The native peptide (L_2_C_3_V_10_C_11_) has the lowest net charge but the highest hydrophobicity and the peptides K_2_W_3_V_10_R_11_ and W_2_R_3_V_10_R_11_ have the same net charge and similar hydrophobicity. The relative hydrophobic moment (μHrel), which reveals the amphiphilicity of the peptides, is positively correlated with amphiphilicity. The results showed that the μHrel value of the designed peptides was decreased gradually, ranging from 0.072 to 0.479. Figure 1 presents an Edmundson helical wheel diagram of the amphiphilic properties of amino acid residues in each peptide. The wheel diagram and the relative hydrophobic moments revealed the designed peptides presented a better balance between the hydrophobic and hydrophilic phases.

**TABLE 1:** Amino acid sequences and physicochemical properties of the peptides.

**AMPs**	**Sequence**	**Theoretical MW**	**Measured MW^a^**	**Net charge**	**H^b^**	**R_t_ (min)^c^**	**μHrel^d^**
L_2_C_3_V_10_C_11_	RLCRIVVIRVCR-NH_2_	1485.92	1484.94	+4	0.667	18.4	0.321
R_2_F_3_W_10_L_11_	RRFRIVVIRWLR-NH_2_	1670.09	1669.12	+5	0.561	15.8	0.479
R_2_W_3_W_10_R_11_	RRWRIVVIRWRR-NH_2_	1752.15	1751.18	+6	0.373	14.3	0.464
K_2_W_3_V_10_R_11_	RKWRIVVIRVRR-NH_2_	1637.06	1636.09	+6	0.289	12.1	0.379
W_2_R_3_V_10_R_11_	RWRRIVVIRVRR-NH_2_	1665.07	1664.10	+6	0.288	11.6	0.324
W_2_K_3_K_10_R_11_	RWKRIVVIRKRR-NH_2_	1666.10	1665.13	+7	0.105	9.8	0.280
K_2_R_3_R_10_K_11_	RKRRIVVIRRKR-NH_2_	1636.07	1635.10	+8	−0.167	9.1	0.072

*^*a*^Molecular weight (MW) was measured by mass spectroscopy (MS). ^*b*^The mean hydrophobicity (H) is the total hydrophobicity (sum of all residue hydrophobicity indices) divided by the number of residues. ^*c*^RP-HPLC retention time (R_*t*_) was measured using a C_18_ RP column (5 μm; 4.6 × 250 nm; Vydac). ^*d*^The relative hydrophobic moment (μHrel) of a peptide is its hydrophobic moment relative to that of a perfectly amphipathic peptide. This yields a better measure of the amphipathicity using different scales. A value of 0.5 indicates that the peptide has about 50% of the maximum possible amphipathicity.*

### Circular Dichroism Spectra

**FIGURE 2:**
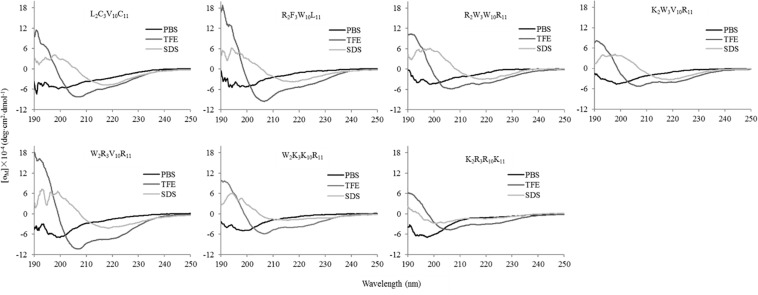
The circular dichroism (CD) spectra of the peptides in different environments. The peptides were dissolved in 10 mM phosphate buffered saline (PBS pH 7.4), 50% trifluoroethanol (TFE), and 30 mM sodium dodecyl sulfate (SDS), respectively. The mean residue ellipticity was plotted against wavelength. The values from three scans were averaged per sample, and the peptide concentrations were fixed at 150 μM.

The secondary structures of peptides in different environments (10 mM PBS, 50% TFE, and 30 mM SDS) were determined by circular dichroism spectra, as shown in Figure 2. All CD spectra were analyzed using SELCON3 program (Sreerama et al., 2000), the results are summarized in Table 2. In PBS, all peptides appeared at a negative peak in the 195–202 nm range and a value near zero at 220 nm, and the unordered content of theses peptides ranged from 51 to 66%, indicating that peptides revealed the presence of discorded conformers in a mimic aqueous environment (Pandey et al., 2010). In SDS, all peptides displayed a positive peak in around 195-197 nm range and a typical negative peak at 217–218 nm, and showed a negative band at around 220 nm, the α-helical content of these peptides ranged from 38 to 49%, the β-strand content of these peptides ranged from 20 to 29%, indicating that these peptides revealed α-helix and β-sheet structure in a mimic microbial membrane environment (Unger et al., 2001). In TFE, the peptides displayed a positive peak at around 191 nm and a negative dichroic bands at approximately 208 and 222 nm, which is consistent with the predominant induction of α-helix conformations in the mimic hydrophobic environment (Chou et al., 2016), the α-helical content of these peptides ranged from 51 to 84%. These data indicated that the α-helical structural characteristic and the propensity to form an amphipathic α-helix of the peptide in microbial membrane-mimetic environments may play an important role in killing bacterial cells.

**TABLE 2:** Circular dichroism data of the peptides in various solutions.

**Peptides**	**PBS**				**SDS**				**TFE**			
	**α-helix**	**β-strand**	**Turn**	**Unordered**	**α-helix**	**β-strand**	**Turn**	**Unordered**	**α-helix**	**β-strand**	**Turn**	**Unordered**
L_2_C_3_V_10_C_11_	2%	10%	25%	65%	38%	24%	21%	24%	51%	21%	17%	18%
R_2_F_3_W_10_L_11_	5%	17%	20%	61%	44%	23%	13%	22%	55%	10%	14%	21%
R_2_W_3_W_10_R_11_	7%	13%	23%	56%	43%	20%	19%	28%	60%	16%	16%	17%
K_2_W_3_V_10_R_11_	11%	14%	15%	66%	49%	23%	15%	20%	84%	10%	11%	9%
W_2_R_3_V_10_R_11_	11%	19%	21%	51%	45%	24%	19%	22%	74%	7%	12%	10%
W_2_K_3_K_10_R_11_	7%	16%	23%	56%	41%	25%	15%	29%	52%	13%	18%	19%
K_2_R_3_R_10_K_11_	7%	21%	21%	58%	40%	29%	16%	21%	60%	10%	20%	13%

### Antimicrobial Activity

The antimicrobial effect of peptides on 12 typical food-pathogens is listed in Table 3. The geometric mean (GM) revealed the inhibitory effect of peptides against the food-pathogens. In Table 3, the GMs of the designed peptides (except W_2_K_3_K_10_R_11_, K_2_R_3_R_10_K_11_) are markedly lower than that of the native peptide (L_2_C_3_V_10_C_11_), indicating that the antimicrobial activities of the peptides R_2_F_3_W_10_L_11_, R_2_W_3_W_10_R_11_, K_2_W_3_V_10_R_11_, and W_2_R_3_V_10_R_1__1_ were higher than that of the native peptide, W_2_K_3_K_10_R_11_ and K_2_R_3_R_10_K_11_. The minimum hemolytic concentration (MHC) of the designed peptides were markedly higher than that of the native peptide (L_2_C_3_V_10_C_11_), indicating that the hemolytic activity of the designed peptides was lower than that of the native peptide. In addition to antimicrobial activity, the design of antimicrobial peptides should also consider the selectivity of peptides to mammalian cells. The selectivity index (SI), calculated as the ratio of MHC to GM, was used a comparison parameter for the safety and efficacy of peptides. The SI values of the designed peptides were evidently higher than that of the native peptide because of their higher MHC values. Specifically, the peptides W_2_R_3_V_10_R_1__1_, K_2_W_3_V_10_R_11_ had the highest SI values, indicating that they had considerably high levels of cell selectivity for pathogens. Table 3 shows that the antibacterial activity and cell selectivity of the designed peptides was higher than that of the native peptide (L_2_C_3_V_10_C_11_), particularly for Gram-negative bacteria.

**TABLE 3:** Antimicrobial activity of the peptides.

	**MIC^a^ (μM)**						
	**L_2_C_3_ V_10_C_11_**	**R_2_F_3_ W_10_L_11_**	**R_2_W_3_ W_10_R_11_**	**K_2_W_3_ V_10_R_11_**	**W_2_R_3_ V_10_R_11_**	**W_2_K_3_ K_10_R_11_**	**K_2_R_3_ R_10_K_11_**
**Gram-negative bacteria**							
*Escherichia coli* ATCC25922	4	2	2	2	2	4	8
*Escherichia coli* UB1005	128	16	16	8	16	256	256
*Salmonella typhimurium* C77-31	>256	64	64	32	32	256	>256
*Salmonella typhimurium* ATCC14028	128	32	64	32	32	128	128
*Salmonella pullorum* C79-13	>256	32	32	8	32	256	256
*Salmonella enteric-subspenterica* CMCC47020	128	64	256	64	128	>256	256
*Salmonella enteric-subspenterica* CMCC50071	64	8	8	8	8	128	>256
*Cronobacter sakazakii* ATCC29544	256	32	64	32	16	256	>256
**Gram-positive bacteria**							
*Listeria monocytogenes* CMCC54004	128	16	1	2	2	128	32
*Bacillus cereus* CMCC6303	64	32	64	16	64	128	128
*Staphylococcus aureus* CMCC26074	>256	256	128	256	64	>256	>256
*Staphylococcus aureus* ATCC25923	>256	256	128	128	256	>256	>256
MHC^b^ (μM)	64	128	128	>256	>256	>256	>256
GM^c^ (μM)	143.7	32.0	30.2	19.0	24.0	170.0	191.8
SI^d^	0.5	4.0	4.2	26.9	21.5	3.0	2.7

*^*a*^The minimum inhibitory concentration (MIC, μM) was determined as the lowest concentration of peptide that inhibited bacterial growth. ^*b*^MHC was the minimum hemolytic concentration that caused 5% hemolysis of human red blood cells (hRBCs). ^*c*^The geometric mean (GM) of the peptide MIC against all bacterial strains was calculated. ^*d*^The selectivity index (SI) is the ratio of the MHC to the geometric mean of MIC. When MIC or MHC is over 256 μM, a value of 512 μM is used to calculate.*

### Hemolytic Activity

**FIGURE 3:**
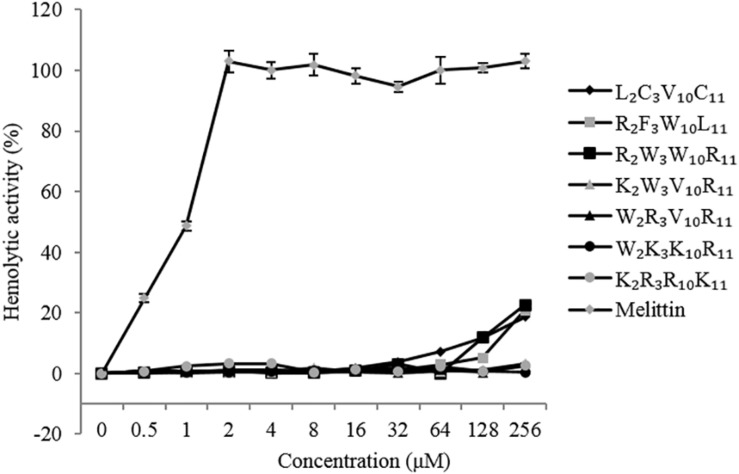
Hemolytic activity of the peptides against human red blood cells (hRBCs).

The hemolytic activity of the peptides was determined by measuring the amount of hemoglobin released by hRBCs (Figure 3). It is desirable that the MHC values of the peptides were as high as possible in comparison to the MIC. At peptide concentrations ranging from 0 to 256 μM, the hemolytic activity of native and designed peptides was significantly lower than that of melittin (positive control). The highest hemolytic activity of the native and designed peptides was only 23% at the concentration of 256 μM, whereas that of melittin was 100% at the concentration of 4 μM. The hemolytic activity of the designed peptides (except K_2_R_3_R_10_K_11_) was similar to that of the native peptide when the peptide concentration was between 0 and 16 μM. Specifically, the hemolytic activity of the peptides K_2_W_3_V_10_R_11_, W_2_R_3_V_10_L_11_, W_2_K_3_K_10_R_11_, and K_2_R_3_R_10_K_11_ were lower than that of the native peptide when the peptide concentration exceeds 16 μM. Overall, the native peptide showed 5% hemolysis at 64 μM (Table 3), the designed peptides caused 5% hemolysis at the minimum concentration of 128 μM, indicating that the hemolytic activity of the designed peptides was lower than that of the native peptide.

### Cytotoxicity

**FIGURE 4:**
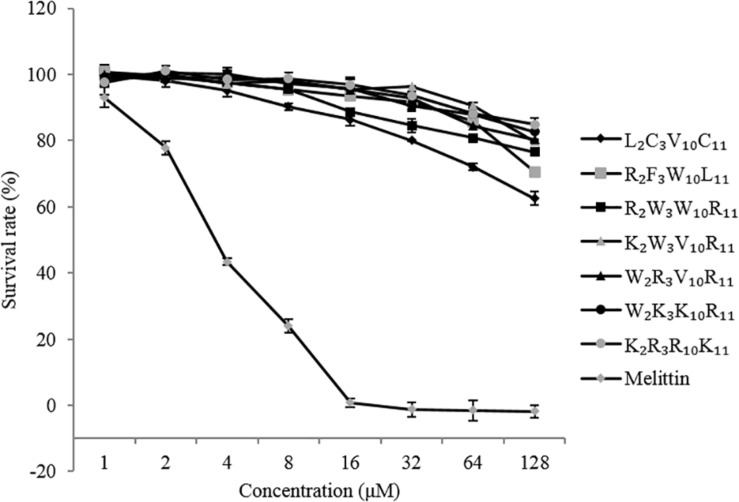
Cytotoxicity of the peptides against EA. hy 926 cells.

The cytotoxicity of the peptides against EA. hy 926 cells is shown in Figure 4. In general, the cytotoxicity of these peptides against EA. hy 926 cells was consistent with their hemolytic activities to hRBCs. The native and designed peptides exhibit significantly lower cytotoxicity than that of melittin (positive control) at peptides concentrations ranging from 1 to 128 μM. The viability of the EA. hy 926 cells decreased with an increase in peptide concentration. The cytotoxicity of melittin reached 100% at the concentration of 16 μM. When peptide concentration reached 128 μM, the EA. hy 926 cell viability of each peptide was as follows: L_2_C_3_V_10_C_11_, 63%; R_2_F_3_W_10_L_11_, 71%; R_2_W_3_W_10_R_11_, 77%; K_2_W_3_V_10_R_11_, 80%; W_2_R_3_V_10_R_11_, 80%; W_2_K_3_K_10_R_11_, 83%; and K_2_R_3_R_10_K_11_, 85%. The designed peptides were less cytotoxic than the native peptide and showed much higher selectivity for EA. hy 926 cells, which is considered desirable.

### Stability

To evaluate the stability of peptides under different salt, heat, and protease conditions, we determined the MICs of the peptides against *Escherichia coli* ATCC25922, because Gram-negative bacteria were sensitive to the designed peptides. Table 4 shows the change in the MICs of peptides under different conditions. All peptides exhibited increased MICs under Na^+^ and heat conditions; in other salt conditions, the MICs of individual peptides increased. Notably, the change in MICs of all peptides remained considerably small, indicating that all peptides were relatively stable in different physiological concentrations of salt and heat. All peptides exhibited no sensitivity to papain. By contrast, all peptides showed sensitivity to trypsin, particularly designed peptides, which had almost no antimicrobial activity. The MIC of K_2_W_3_V_10_R_11_ varied markedly, reaching 128 and 256 μM following treatment with pepsin and protease k, respectively. The MICs of R_2_F_3_W_10_L_11_ and R_2_W_3_W_10_R_11_ reached 128 μM under proteinase k conditions.

**TABLE 4:** MIC^a^ values of peptides in the presence of salts, heat and proteases.

**AMP**	**Control**	**NaCl^b^**	**KCl^b^**	**MgCl_2_^b^**	**CaCl_2_^b^**	**NH_4_Cl^b^**	**Heat^c^**	**Pepsin^d^**	**Trypsin^d^**	**Papain^d^**	**Proteinase k^d^**
L_2_C_3_V_10_C_11_	4	16	4	4	8	4	8	16	64	16	32
R_2_F_3_W_10_L_11_	2	16	8	2	8	4	32	16	>256	8	128
R_2_W_3_W_10_R_11_	2	4	2	2	2	2	8	32	>256	4	128
K_2_W_3_V_10_R_11_	2	8	2	4	8	2	8	128	>256	2	256
W_2_R_3_V_10_R_1__1_	2	4	2	4	8	2	4	32	>256	2	8
W_2_K_3_K_10_R_11_	4	8	4	4	4	4	16	16	>256	4	8
K_2_R_3_R_10_K_11_	8	32	8	8	16	8	16	32	>256	8	8

*^*a*^The minimum inhibitory concentration (MIC, μM) was determined as the lowest concentration of peptide that inhibited bacterial growth. ^*b*^The final concentrations of NaCl, KCl, NH_4_Cl, MgCl_2_, ZnCl_2_, CaCl_2_, and FeCl_3_ were 150 mM, 4.5 mM, 6 μM, 1 mM, 8 μM, 2 mM, and 4 μM, respectively. ^*c*^The temperature is 100°C. ^*d*^The final concentrations of trypsin, pepsin, papain and protease k were 1 mg/mL.*

### Antibacterial Mechanism Study

With the biological activity of the aforementioned designed peptides considered, the peptides R_2_F_3_W_10_L_11_, R_2_W_3_W_10_R_11_, K_2_W_3_V_10_R_11_, and W_2_R_3_V_10_R_1__1_ were selected to explore the antibacterial mechanism of AMPs.

#### Outer Membrane Permeability

**FIGURE 5:**
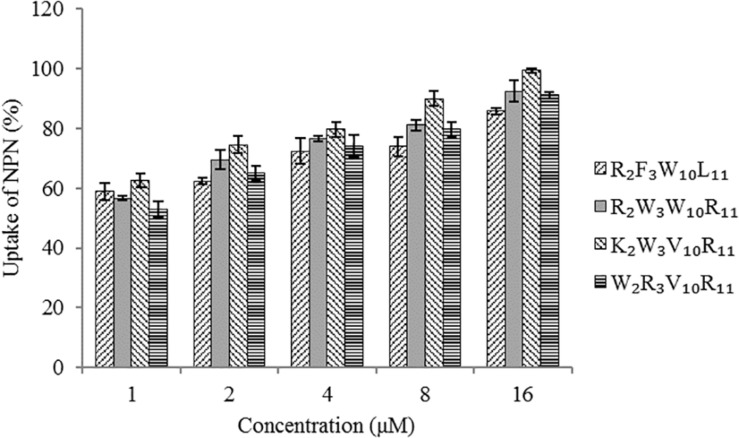
Outer membrane permeability of the peptides. The uptake of NPN of *Escherichia coli* UB1005 in the presence of peptides at 1 × MIC was determined using the fluorescent dye NPN assay at an excitation wavelength of 350 nm and an emission wavelength of 420 nm.

The hydrophobic fluorophore NPN is normally excluded by the outer membrane, but it is taken up and exhibits increased fluorescence intensity upon permeabilization of the outer membrane. Figure 5 shows the effect of peptides on the outer membrane permeability of *Escherichia coli* UB1005 by measuring NPN uptake. The four designed peptides increased the outer membrane permeability of *Escherichia coli* UB1005 in a dose-dependent manner at concentrations from 1 to 16 μM. Under the same concentration, the NPN uptake caused by the peptide K_2_W_3_V_10_R_11_ was higher than those of the other three peptides, indicating that the outer membrane permeability attributed to the peptide K_2_W_3_V_10_R_11_ was the largest, and the destruction of the outer membrane was the largest, which was related to the antibacterial activity of the peptide K_2_W_3_V_10_R_11_ to *Escherichia coli* UB1005.

#### Inner Membrane Permeability

**FIGURE 6:**
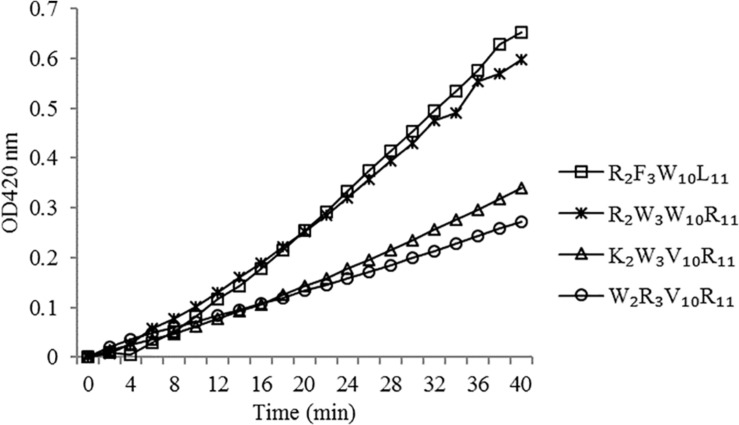
Inner membrane permeability of the peptides. The uptake of ONPG of *Escherichia coli* UB1005 treated with peptides at 1 × MIC was determined at an excitation wavelength of 420 nm.

The peptides induced permeabilization of the inner membrane, ONPG entered the cytoplasm and was degraded by β-galactosidase into o-nitrophenol, which produced absorbance at 420 nm. We investigated the inner membrane permeabilization induced by the peptides by measuring the ONPG uptake (Figure 6). The peptides induced an increase in inner membrane permeability in a time-dependent manner at 1× MIC. The inner membrane permeability of the peptide R_2_F_3_W_10_L_11_ was similar to that of the peptide R_2_W_3_W_10_R_11_ and markedly higher than those of the other two peptides. The peptides, arranged in descending order with respect to membrane permeability, were as follows: R_2_F_3_W_10_L_11_ > R_2_W_3_W_10_R_11_ > K_2_W_3_V_10_R_11_ > W_2_R_3_V_10_R_1__1_.

#### Cytoplasmic Membrane Depolarization

**FIGURE 7:**
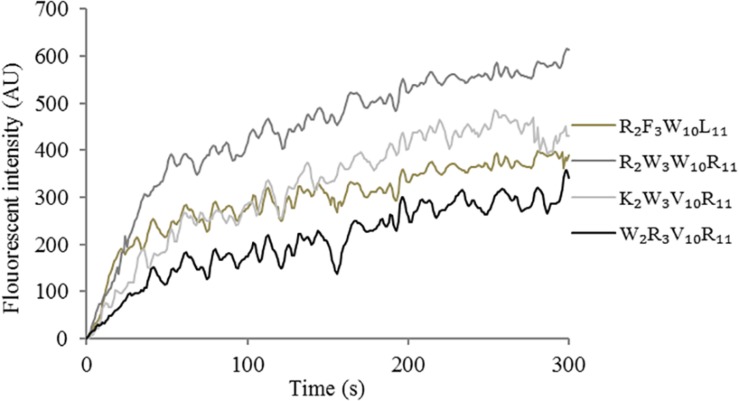
Cytoplasmic membrane depolarization of *Escherichia coli* UB1005 treated with peptides at 1 × MIC was assessed by measuring the release of the membrane potential-sensitive fluorescent dye diSC_3_-5 at an excitation wavelength of 622 nm and an emission wavelength of 670 nm from 0 s to 300 s.

The depolarization effect of peptides at 1 × MIC on the *Escherichia coli* UB1005 cytoplasmic membrane was evaluated by measuring the fluorescence intensity of diSC_3_-5. Upon the permeabilization and disruption of the outer and inner membrane, the electrical potential of the cytoplasmic membrane will change. The cationic dye is concentrated in the cytoplasmic membrane under the influence of the internally negative membrane potential. In Figure 7, the peptides cause a rapid increase in fluorescence intensity, reflecting cytoplasmic membrane depolarization. The cytoplasmic membrane of the peptide R_2_W_3_W_10_R_11_ was depolarized faster and stronger, compared with other peptides over 300 s. The peptides, arranged in descending order with respect to cytoplasmic membrane depolarization, were as follows: R_2_W_3_W_10_R_11_ > K_2_W_3_V_10_R_11_ > R_2_F_3_W_10_L_11_ > W_2_R_3_V_10_R_1__1_. The increasing fluorescence intensity indicated that cytoplasmic membrane depolarization exhibited time dependence.

#### Flow Cytometry

**FIGURE 8:**
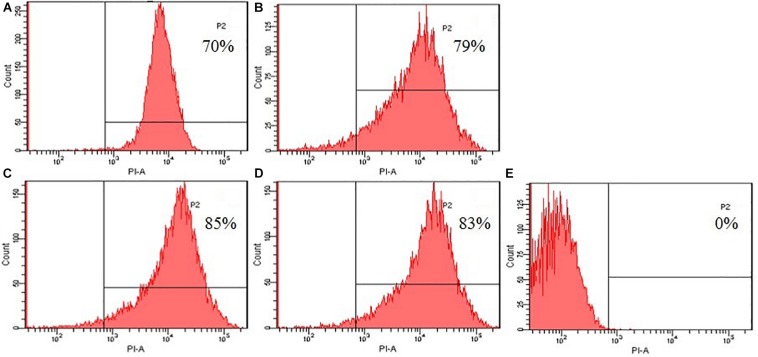
Destruction of cytomembrane integrity treated by peptides. The uptake of PI of *Escherichia coli* ATCC25922 treated with peptides at 1 × MIC was determined at 4°C for 30 min. **(A)** R_2_F_3_W_10_L_11_; **(B)** R_2_W_3_W_10_R_11_; **(C)** K_2_W_3_V_10_R_11_; **(D)** W_2_R_3_V_10_R_1__1_; **(E)** Control.

To further evaluated the broken effect of AMPs on bacteria *Escherichia coli* ATCC25922 cell membranes, we detected a PI fluorescent signal by flow cytometry (Figure 8). PI fluorescently stained the nucleic acids of cells when cells suffered disruption of cytoplasmic membrane integrity. The results showed that only 0.1% of the fluorescent signals were observed in the control (no peptide), indicating that most cell membranes were intact. The PI fluorescent signals were 70, 79, 85, and 83% at the concentration of 1 × MIC, suggesting that most bacterial cell membranes were destroyed, the peptide K_2_W_3_V_10_R_11_ and W_2_R_3_V_10_R_1__1_ were more destructive.

#### Electron Microscopic Characterization

**FIGURE 9:**
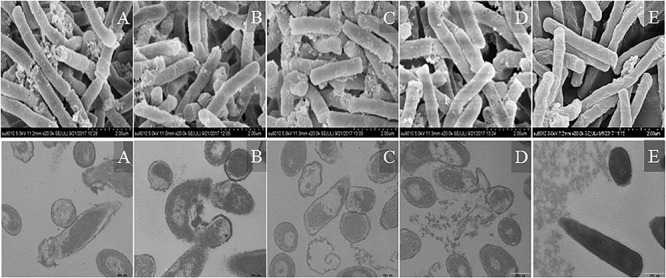
SEM and TEM micrographs of *Escherichia coli* ATCC25922 cells treated with peptides at 1 × MIC for 1 h. **(A)** R_2_F_3_W_10_L_11_; **(B)** R_2_W_3_W_10_R_11_; **(C)** K_2_W_3_V_10_R_11_; **(D)** W_2_R_3_V_10_R_1__1_; **(E)** Control.

Morphological changes in *Escherichia coli* ATCC25922 cells treated with peptides were observed by SEM and TEM (Figure 9). The cells treated with peptides at the concentration of 1 × MIC showed evident membrane damage compared with the control (no peptide). The membrane surface was complete and smooth without obvious holes. The surface of the bacterial cell membrane surface became rough and wrinkled, atrophied, and fractured. The cells produced significant leakage and fracture. Intracellular changes of bacteria in *Escherichia coli* ATCC25922 cells were observed by TEM. The control (no peptide) exhibited an intact and smooth surface, distinct cell wall, and evenly distributed cell contents. The cells treated with AMPs at the concentration of 1 × MIC showed apparent intracellular alteration; the distribution of the intracellular contents was no longer uniform and separated from the membrane. The cell membrane was destroyed, revealing holes, and the intracellular content was leaked, resulting in cells death.

## Discussion

Owing to the increasing resistance of pathogens to antibiotics, AMPs emerged as a potential alternative to antibiotics for AMPs kill bacteria by destroying the integrity of cell membranes, greatly reducing the likelihood of bacterial resistance. The objective of the present study was to develop bactenecin derivatives with higher antimicrobial activity, broader antibacterial spectrum, and lower cytotoxicity. The biological activity of AMPs is linked to their physicochemical properties, such as net charge, hydrophobicity, amphipathy and so on. In this study, arginine and lysine were introduced in the designed peptides to increase the electrostatic interaction with the negatively charged surface of bacterial membranes (Ma et al., 2011), tryptophan and phenylalanine were introduced in the designed peptides to ensure a more efficient interaction with the interface of lipid bilayers (Kyoungsoo et al., 2002).

Most AMPs possess a positive charge, whereas bacteria have a negative charge on their cell membranes. Thus, AMPs can play an antibacterial role by combining with bacterial cell membranes via electrostatic attraction. The introduction of a positive charge into the antimicrobial peptide can lead to an increase in its antimicrobial activity, which may be due to the improved stability of the molecular structure (e.g., α-helix) of the peptide. No positive correlation exists between the number of positive charges and antibacterial activity. In the current study, the peptides arranged in ascending order with respect to net charge, as follows: L_2_C_3_V_10_C_11_ (+4) < R_2_F_3_W_10_L_11_ (+5) < R_2_W_3_W_10_R_11_ (+6) = K_2_W_3_V_10_R_11_ (+6) = W_2_R_3_V_10_R_11_ (+6) < W_2_K_3_K_10_R_11_ (+7) < K_2_R_3_R_10_K_11_ (+8). The antibacterial activity of peptides initially increased and then decreased as the net charge of the peptides increased (Table 3). On the one hand, the positive charge keep AMPs adherent to the surface of cell membranes, reducing its ability to penetrate the membranes; on the other hand, this occurrence may increase electrostatic repulsion among peptide molecules; when the electrostatic repulsion is greater than the electrostatic attraction between AMPs and cell membranes, aggregation of antibacterial peptide molecules on cell membranes is prevented (Matsuzaki, 1999). The two cysteine residues form a disulfide bond to make the native peptide a loop molecule, which reduces the tendency to form amphiphilic helical structures, leading to lower antimicrobial activity. The antibacterial activities of the peptides K_2_W_3_V_10_R_11_ and W_2_R_3_V_10_R_11_ were significantly higher, compared with the other peptides. At a net charge of +6, the peptides K_2_W_3_V_10_R_11_ and W_2_R_3_V_10_R_11_ exhibited the highest (and similar) antibacterial activity. The reason could be that the hydrophobic valine residue in position 10 is more likely to form a stable α-helical structure due to its relatively small side chain; α-helical structure plays an important role in antimicrobial activity of antimicrobial peptides. Furthermore, the consecutive same positively charged amino acids may result in steric hindrance and charge repulsion, causing the disruption of helix formation, leading to a significant reduction in antimicrobial activity (Zhu et al., 2015).

Hydrophobicity is another important factor that defines the antimicrobial potency, and the hydrophobicity of these substituted amino acid residues decreased in the order Val > Trp > Lys > Arg, consistent with the order of antimicrobial activity of these peptides. The hydrophobic groups of amino acids allow AMP molecules to form a polysome by the hydrophobic action in the solution and played an improved role in the cell membrane. On the one hand, low hydrophobicity could weaken the ability of AMP molecules to bind to cell membranes; while high hydrophobicity could cause the AMP molecules to aggregate. Extreme cationicity and hydrophobicity have been found to hamper selective electrostatic interactions and favor interactions with zwitterionic phospholipids, concomitantly and inadvertently resulting in an increase in cytotoxicity and loss of cell selectivity (Zhang et al., 2010; Huang Y. B. et al., 2011). The hemolytic activity and cytotoxicity of AMPs limit their application. The higher the hydrophobicity, the greater the hemolytic activity and cytotoxicity of AMPs (Zhang et al., 2010). Compared with melittin, the native and designed peptides showed lower hemolytic activity and cytotoxicity (Figures 3, 4). Overall, the designed peptides had lower hemolytic activity and cytotoxicity than the native peptide. The hemolytic activity and cytotoxicity of the peptides bactenecin, R_2_F_3_W_10_L_11_, and R_2_W_3_W_10_R_11_ were higher than those of the other peptides. The reason could be that these peptides exhibited relatively higher hydrophobicity and relative hydrophobic moment (amphiphilicity) because they contain more hydrophobic amino acids residues. Previous studies have shown that hemolytic activity is positively correlated with the side chain hydrophobicity of the substituting amino acid residue, peptide hydrophobicity, amphipathicity (Hawrani et al., 2008). Furthermore, the disruption of the hydrophobic face by the introduction of a charged residue was reported to significantly reduce hemolytic activity and cytotoxicity without deteriorating antimicrobial activity.

Amphipathicity also appear to have influence on the antimicrobial activity of AMPs. Amphipathic residue arrangement is important for the activity of α-helical AMPs because the polar region allows the molecules to assemble on the membrane through electrostatic interaction with the negatively charged head groups of phospholipids, and then the non-polar region would lead to the formation of transient pores or channels through hydrophobic interactions, causing increased permeability and loss of barrier function of target cells (Sato and Feix, 2006). If the peptides display helix formation in the membrane environment, they would have greater affinity for the anionic membrane and have enhanced incorporation into lipid membranes, further increasing the antimicrobial activity of AMPs (Ahmad et al., 2013). Amphiphilicity affects the conformation of AMPs in different environments. Circular dichroism spectroscopy can provide useful information about the conformations and structural changes of the peptides in different environments. Most AMPs adopt the α-helical and/or β-sheet structures (Wang et al., 2013; Wiradharma et al., 2013), which are beneficial for the interaction between antimicrobial peptides and cell membranes (Henzler Wildman et al., 2003; Dong et al., 2012), and this phenomenon is particularly evident from CD spectral studies. The amphipathic AMPs undergo important conformational changes from random coils in aqueous solution to helical structures induced by anisotropic environments. Our results indicated that the designed peptides appear to present higher helical content than the native peptide. In 10 mM PBS, the native and designed peptides showed a disordered structure in a mimic aqueous environment; in 50%TFE, these peptides exhibited an α-helical structure in a mimic microbial membrane environment; in 30 mM SDS, these peptides adopted a mixed α-helix and β-sheet structure in the SDS-micellular membrane mimicking environment. Given the structural data from the CD studies, it is clear that the amphipathicity structure significantly increases the propensity for α-helix formation in the bacterial membrane-mimicking environments, which is correlated with the antimicrobial activity of AMPs. The presence of the basic residue in the peptide sequence could also contribute to a stabilizing effect to the helical structure by increasing the helical macrodipole and providing an extra hydrogen bonding between lateral chains especially arginine. Cation–π interactions occur between basic residues (lysine and arginine) and aromatic residues (tryptophan and phenylalanine) and are important for peptide self-association within membranes and facilitate deeper insertion into membranes by shielding cationic side chains (Gallivan and Dougherty, 1999).

Studies on numerous natural peptides have shown that some cations (Na^+^ and Mg^2+^) may affect the activity of peptides relying on the interruption of the electrostatic attraction between the positively charged peptides and the negatively charged membranes and the competition for membrane binding between the peptides and cations (Wu et al., 2008; Mcdonnell et al., 2012). Electrostatic interaction between cationic peptides and cell membranes is necessary for peptides to kill bacteria (Saardover et al., 2012). The higher the ionic strength, the lower the electrostatic adsorption between the cationic AMPs and the lipid bilayers of the bacterial anionic cell membrane, causing a decrease in antibacterial activity (Huang J. et al., 2011). However, in this study, cations caused a 2-4-fold change in MIC values, indicating that the designed peptides still maintained a relatively desirable active state. The designed peptides showed salt-resistance, possibly owing to their high net charge, which can overcome cationic masking by anions in salts and promote peptide interaction with the cell membranes (Sugiarto and Yu, 2007). The designed peptides maintained high antibacterial activity after treatment at 100°C for 1 h, suggesting that the designed peptides showed good thermal stability. This finding is consistent with the results of a recent study by Ma et al. (2012). The reason may be that the peptides present a disordered structure in mimicking the aqueous environment and forms α-helical structure or β-sheet structure in mimicking the membrane environment. The antibacterial activity of antimicrobial peptides treated with enzymes decreased to varying degrees. The antimicrobial peptides treated by the enzyme showed antibacterial activity reduced to varying degrees, potentially resulting from the different amounts of arginine. The stability of peptides to enzymes was slightly low. In the presence of salt and heat, the peptides generally showed high antibacterial activity, exhibiting potential application for further development of antibacterial agents.

The action mechanism of AMPs depends on their damage to cell membranes integrity via barrel-stave or toroidal-pore mechanisms, or induce ‘micellization’ by carpet mechanisms (Brogden, 2005). The bacterial cell outer membranes consist of negatively charged teichoic acid, or LPS, hydroxy-phospholipids such as phosphatidylserine, cardiolipin and phosphatidylglycerol, which promote interaction with cationic peptides (Teixeira et al., 2012; Mai et al., 2017). The cationic antimicrobial peptides are electrostatically attracted by the negatively charged bacterial surface layers and get itself embedded into the hydrophobic regions of the lipid membranes, leading to destruction of the organism by formation of transient pores or channels, destabilization of membrane equilibrium, or penetration into the cell, causing increased membrane permeability, membrane depolarization, cell cytoplasmic membrane lysis, and cytoplasmic leakage; ultimately, this process leads to cell death (Ong et al., 2014). In this study, Gram-negative bacteria were more sensitive to the designed peptides and more clearly observed, *Escherichia coli* ATCC25922 cells were used as an indicator strain. This is because the cell wall of Gram-positive bacteria is relatively thick, containing a large amount of peptide-glycan and teichoic acid, while the cell wall of Gram-negative bacteria is relatively thin, containing a large number of LPS, phospholipids and a small amount of peptide-glycan, which leads to the loose outer membrane structure of Gram-negative bacteria, making it easier for antimicrobial peptides to embed into the cell membrane. The results of this study indicated that the designed peptides could rapidly damage the bacterial cell outer membrane. Moreover, the designed peptides at 1 × MIC were able to permeabilize the inner membrane. As a result of cell membrane rupture, cytoplasmic membrane potentially increased, leading to cytoplasmic leakage and ultimately, cell death. The results of SEM and TEM confirmed the strong membrane rupture ability of the designed peptides.

## Conclusion

We designed and synthesized cationic AMPs based on the native peptide (bactenecin, L_2_C_3_V_10_C_11_) and then evaluated their bioactivity. These peptides exhibited a disordered structure in an aqueous solution but folded into an α-helical structure and/or β-sheet structure in a membrane environment. The designed peptides exhibited stronger antimicrobial activity, lower level of hemolysis, and cytotoxicity than those of the native peptide, especially K_2_W_3_V_10_R_11_ and W_2_R_3_V_10_R_11_. At MIC, the peptides permeabilized the bacterial cell outer and inner membrane, and depolarized the cytoplasmic membrane, disrupting the integrity of the membrane. This disruption caused cytoplasm leakage, leading to cell death. All these results indicated that the designed peptides exerted an antibacterial effect against food-pathogens. The findings of this study also provide a rationalization for the design of antibacterial peptides, which is important for the future development of antimicrobial agents.

## Data Availability Statement

All datasets generated for this study are included in the article/supplementary material.

## Author Contributions

CS and HW designed the study. LC, LL, CJ, and LG provided advice on experimental design and data analysis. LG and SP collected and assembled the data. CS wrote the manuscript. CS and MH revised the manuscript. JH and ZJ supervised the study. All authors reviewed and commented on the manuscript.

## Conflict of Interest

LC and LL were employed by Beijing Sanyuan Foods Co., Ltd.

The remaining authors declare that the research was conducted in the absence of any commercial or financial relationships that could be construed as a potential conflict of interest.
